# Intention to Use Postpartum Contraceptive and Its Determinants in Sub-Saharan Africa: Systematic Review and Meta-Analysis

**DOI:** 10.1089/whr.2023.0059

**Published:** 2023-12-15

**Authors:** Natnael Atnafu Gebeyehu, Kirubel Dagnaw Tegegne

**Affiliations:** ^1^ Department of Midwifery, College of Medicine and Health Science, Wolaita Sodo University, Sodo, Ethiopia.; ^2^ Department of Comprehensive Nursing, College of Medicine and Health Science, Wollo University, Wollo, Ethiopia.

**Keywords:** intention, postpartum period, contraception, systematic review, meta-analysis, sub-Saharan Africa

## Abstract

**Background:**

The postpartum period is a critical moment for the delivery of family planning services. However, the utilization of family planning among women in sub-Saharan Africa is not optimal. Therefore, the current study aims to assess the intention to use postpartum contraception and its related determinants in the sub-Saharan African setting.

**Methods:**

This study utilized a comprehensive search strategy that involved searching several databases, including PubMed, Scopus, EMBASE, Science Direct, Google Scholar, and online research institutional repository homes. Data extraction was performed using Microsoft Excel, and statistical analysis was conducted using STATA software (version 14). To assess publication bias, a forest plot, Begg's rank test, and Egger's regression test were employed. Heterogeneity was evaluated using the *I^2^* statistic, and an overall estimated analysis was conducted. In addition, sensitivity analysis was performed to examine the impact of each study on the overall estimate. Meta-regression analysis was conducted to identify potential sources of heterogeneity. Finally, the pooled odds ratio (OR) for associated factors was calculated.

**Result:**

After reviewing 1,321 articles, 14 studies were deemed eligible for inclusion in this meta-analysis. The final analysis comprised a total of 39,936 study participants. The overall intention to use postpartum contraception in sub-Saharan Africa was found to be 62.21% (95% confidence interval [CI]: 55.532–68.875). In subgroup analysis, the highest prevalence of intention was observed in Ethiopia (66.71%; 95% CI: 50.36–83.05), while the lowest prevalence was reported in Ghana (59.39%; 95% CI: 50.22–68.57). The intention to use contraception was found to be 67.22% (95% CI: 62.37–72.07) and 54.53% (95% CI: 46.61–62.45) for institutional and community-based studies, respectively. Maternal educational status (OR = 1.22; 95% CI: 1.09–1.38) and husbands' approval of contraceptive use (OR = 2.395; 95% CI: 1.256–4.567) were identified as predictors of intention to use postpartum contraception.

**Conclusion:**

In conclusion, the results of our study show a comparatively low intention toward the use of postpartum contraception, in contrast to findings reported in other countries. As such, we recommend that stakeholders prioritize maternal education and encourage male partner involvement in family planning decisions.

## Introduction

The primary cause of mortality and morbidity among women of reproductive age is attributed to complications arising from pregnancy and childbirth.^1^ In 2017, the global maternal mortality rate was estimated to be 295,000, with 94% of these deaths occurring in underdeveloped regions. Sub-Saharan Africa alone accounted for ∼66% of maternal mortality, and a significant proportion of these fatalities could have been prevented.^2^ Family planning is widely recognized as a critical approach to reducing maternal and neonatal morbidity and mortality.^3^

In countries with high birth rates, the promotion of family planning has been found to significantly reduce maternal mortality by 32% and child fatalities by nearly 10%.^4^ Postpartum family planning (PPFP) is a strategy employed in the first year following childbirth to prevent unwanted pregnancies and those that are spaced too closely together.^5–7^ PPFP entails the initiation and use of contraception within a year of giving birth.^8^ Despite the potential benefits of PPFP, the low utilization of family planning during the postpartum period has resulted in 80 million unplanned pregnancies worldwide each year.^9^ In sub-Saharan Africa, 40% of postpartum mothers have an unmet need for family planning.^10^ Early initiation of family planning within a year of birth can help prevent these negative outcomes, as two-thirds of maternal and newborn mortality occur during the postpartum period.^11–13^

Short interpregnancy intervals have been linked to a variety of negative health outcomes, such as chronic undernourishment, developmental impairment, miscarriage, stillbirth, induced abortion, preterm birth, low birth weight, neonatal deaths, and maternal deaths, as evidenced by previous studies.^14–18^ In light of these findings, the World Health Organization (WHO) has recommended a minimum of 24 months between successive pregnancies.^19^ A considerable proportion of women who underwent delivery subsequently gave birth to another child within 24–35 months, constituting 35% of the total population. Furthermore, 21% of mothers experienced childbirth within the same timeframe following the birth of their most recent child.^20^ Therefore, the postpartum period is crucial in this context, particularly for initiating contraception and promoting healthy pregnancy spacing.^21–23^

The determination of a woman's intention to use a contraceptive method is a crucial factor in comprehending her future demands and enhancing her likelihood of achieving her objectives.^24^ As it is widely recognized that intentions have a significant impact on behavior, behavioral intentions are employed to assess the efficacy of programs.^25^ Furthermore, intentions are utilized to anticipate the potential requirement for family planning services.^26^ The use of family planning and the intention to use it are the most effective approaches to reducing family size and preventing unintended pregnancies.^27^ In sub-Saharan Africa, women of reproductive age continue to face challenges related to high fertility rates, abortion, and unintended pregnancies.^28^

It has been projected that by the year 2020, an estimated 120 million mothers residing in the most impoverished nations across the globe will have adopted contraceptive measures, as per the objective declared at the London summit.^29^ To achieve the Sustainable Development Goal (SDG3) of ensuring universal access to sexual and reproductive health care services for all individuals by 2030,^30^ it is imperative to establish widespread availability of such services. Nevertheless, the employment of contraceptive methods continues to be limited, particularly in sub-Saharan Africa.^31^

Numerous studies have provided evidence that various sociodemographic factors, including marital status, place of residence, prior parenthood, age, and religious affiliation,^32,33^ exert a significant influence on an individual's inclination to adopt contraceptive measures. In addition, these factors encompass preferences for fertility,^32^ parity,^32^ procreative aspirations,^34^ and misconceptions regarding contraceptive methods.^35^ The consideration of these factors is crucial in assessing an individual's propensity to use contraception, thereby providing empirical support for intervention strategies in sub-Saharan African nations.^36^

Despite the extensive research conducted on postpartum women's contraceptive intentions, there is a dearth of literature on the subject among sub-Saharan African women. Thus, the aim of this systematic review and meta-analysis study is to determine the pooled estimate of intention to use postpartum contraceptives and its determinants among women in sub-Saharan Africa. The findings of this study will provide valuable insights for family planning providers and other stakeholders to address gaps in the intention to use postpartum contraceptives and develop operational plans. This information is crucial in ensuring that every childbearing woman has access to appropriate contraceptive options.

## Methods

### Data Synthesis and Reporting

This study involved the analysis of data derived from a singular measurement outcome, specifically postpartum contraceptive intention. The results were presented through the use of tables, textual descriptions, and a forest plot. Adhering to the Preferred Reporting Items for Systematic Reviews and Meta-Analyses (PRISMA) checklist guideline, a systematic review and meta-analysis were conducted to evaluate the overall intention toward postpartum contraceptive use and its associated factors in sub-Saharan Africa^37^ (Supplementary Table S1). The review protocol was registered with PROSPERO, an international prospective register of systematic reviews, and was assigned the identification number CRD42022357917.

### Search strategy

This study conducted a systematic search for articles on the intention to use postpartum contraceptives in sub-Saharan Africa, using various international online databases such as Pub Med, Science Direct, Scopus, EMBASE, and Google Scholar. In addition, gray literature was retrieved from the institutional research repository of Addis Ababa University. The search was conducted using a set of predetermined keywords and search terms, including “Contraception,” “Family planning,” “Contraceptive,” “Utilization,” “Use,” “Postpartum women,” “Postpartum period,” “Post-delivery,” “Puerperal period,” “intention,” “desire,” and “Sub-Saharan Africa.” Boolean operators such as “OR” and “AND” were employed to combine and separate the search phrases. The Population Exposure Controls and Outcome (PECO) searching guidelines were utilized to perform the search strategy and retrieve relevant articles from the aforementioned databases. The search was conducted between June 1, 2022, and July 10, 2022.

### Study outcome

#### The intention of postpartum contraceptive use

This pertains to the inclination of all women who were identified within a year of parturition and who expressed a desire to utilize any form of contemporary contraception in the future, despite not currently employing any at the time of the survey.^38^

#### Modern contraception

This investigation identifies modern contraceptive methods as comprising female sterilization, male sterilization, intrauterine contraceptive devices, injectable, implants, pills, male condoms, female condoms, and emergency contraception.

#### Sub-Saharan Africa

The countries located in the sub-Saharan region of Africa have been identified and documented by both the United Nations Statistics Division^39^ and the World Bank.^40^

### Inclusion and exclusion criteria

This meta-analysis included studies conducted in sub-Saharan African nations, published in English, and possessing full-text availability for search. Qualitative studies, research from developed countries, duplicated sources, and articles lacking complete text were excluded. The COCOPOP (Condition, Context, and Population) paradigm was employed to determine the eligibility of included articles. Specifically, the study population (POP) consisted of postpartum women, with the condition (CO) being the intention to use postpartum contraceptives, and the context (CO) limited to studies conducted in sub-Saharan Africa.

### Quality assessment

In this study, the standard of research was evaluated by two independent authors, namely N.A.G. and K.D.T., using the Joanna Briggs Institute (JBI) standardized quality appraisal checklist^41^ (Supplementary Table S2). The critical analysis checklist comprised eight parameters, each with options of yes, no, unclear, and not applicable.

These parameters were designed to address the following questions: (1) Were the criteria for inclusion in the sample clearly defined? (2) Was a detailed description provided for the study subjects and setting? (3) Was the exposure measured result valid and reliable? (4) Were the main objective and standard criteria used to measure the event? (5) Were confounding factors identified? (6) Were strategies to affect confounding factors stated? (7) Were the results measured accurately and dependably? and (8) Was the statistical analysis appropriate? The studies were deemed to be of low risk when they scored 50% or higher on the quality assessment indicators.

An initial assessment of this instrument was carried out among a group of skilled health care professionals and academics, indicating that the tool demonstrated facial validity and was well received, easy to use, and efficient in terms of administration. The preliminary results of this exploratory study are encouraging. Nevertheless, to assess its other clinometric properties, particularly its construct validity and inter-rater reliability, this instrument must undergo further examination in a more extensive investigation.

### Risk of bias assessment

Using the bias assessment tool established by Hoy et al.^42^ (Supplementary Table S3), which comprises 10 items that evaluate four domains of bias, as well as internal and external validity, two authors (N.A.G. and K.D.T.) conducted independent evaluations of the included articles for potential bias. The initial four items (items 1–4) appraise the presence of selection bias, nonresponse bias, and external validity. The remaining six items (items 5–10) assess the existence of measurement bias, analysis-related bias, and internal validity.

The agreement between the two reviewers was assessed using their actual agreement and agreement that was not just a coincidence (Kappa). A Kappa value of 0 is regarded as having poor agreement, 0.01 to 0.02 as having only a small agreement, 0.21 to 0.4 as having a fair agreement, 0.41 to 0.60 as having a moderate agreement, 0.61 to 0.80 as having a large agreement, and 0.81 to 1.00 as having a practically perfect agreement.

In this review, a nearly perfect agreement was found, with the real agreement beyond chance falling between 0.88 and 1. Studies were categorized as having a “low risk of bias” if they responded affirmatively to eight or more of the 10 questions. Studies were deemed to have a “moderate risk” if they answered positively to six to seven of the 10 questions, while studies were classified as having a “high risk” if they responded positively to five or fewer of the 10 questions.

### Data extraction

The process of data extraction and analysis was conducted through the utilization of STATA 14 software and a Microsoft Excel spreadsheet from 2016, respectively. By a standardized JBI data extraction format, two authors (N.A.G. and K.D.T.) independently extracted all relevant data. As this study lacked a paper form, the data automation tool was not employed, and manual data extraction was used. The extracted data included the first author's name, the year of publication, the study region, the study setting, the study design, sample size, the prevalence of the intention to use contraception, the unadjusted odd ratio for variables, and the quality of each article.

### Data analysis

This study involved the extraction of relevant data from a Microsoft Excel spreadsheet, which was subsequently exported to STATA software version 14 for analysis. A meta-analysis was conducted using a weighted inverse variance random-effects model to generate a pooled odds ratio (OR). The presence of heterogeneity was assessed visually through a forest plot, which was then used to estimate the pooled estimate of PPFP intention. Subgroup analyses were conducted based on country, sample size, and study setting.

Sensitivity analysis was employed to evaluate the impact of individual studies on the overall prevalence estimate derived from the meta-analysis. The funnel plot was utilized to examine potential publication bias, and Begg's and Egger's regression tests were employed to assess it more objectively. To control for publication bias, the trim-and-fill method proposed by Duval and Tweedie was used.^43^ Cochran's Q χ^2^ test and *I^2^* statistics were used to test for heterogeneity, estimate the amount of total/residual heterogeneity, and measure variability caused by heterogeneity, respectively.^44^ In addition, a univariate meta-regression analysis was conducted to examine the effects of sample size and publication year variations on between-study heterogeneity.^45^

## Results

### Search results and study characteristics

**FIG. 1.**
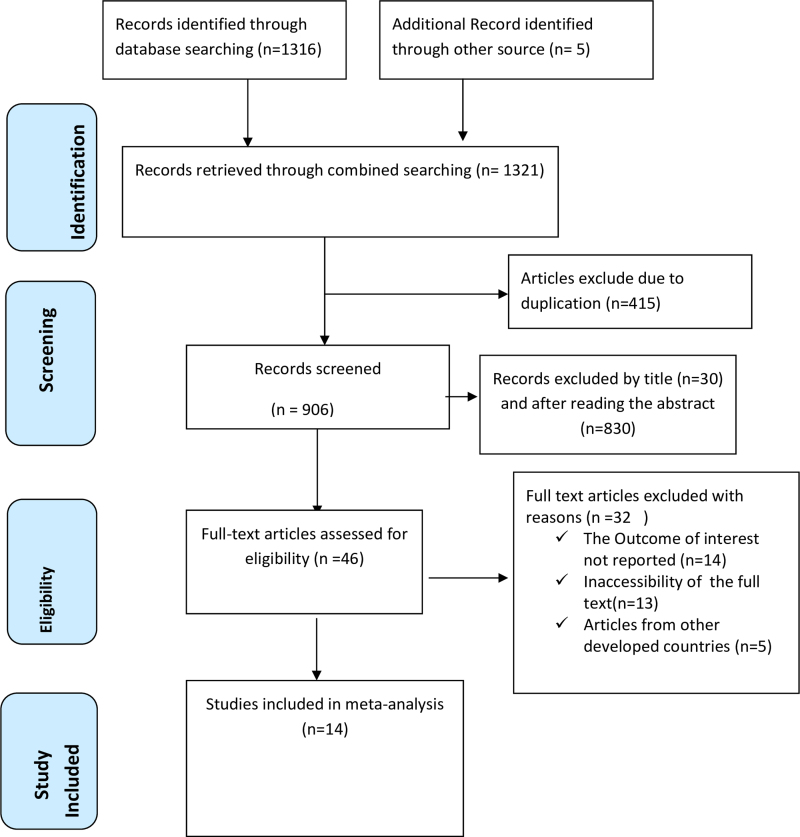
A PRISMA diagrammatic presentation used to show the selection of studies for safe abortion and client satisfaction in Ethiopia. The inclusion criteria were variation of title and abstracts, place of study (sub-Saharan Africa), presence of full abstract, and reporting different results. Studies were excluded if qualitative research, from developed country, duplicated and lacked complete text. PRISMA, Preferred Reporting Items for Systematic Reviews and Meta-Analyses.

A total of 1,321 articles were retrieved from various international online databases, including PubMed, Scopus, EMBASE, Science Direct, and Google Scholar, as well as gray literature from the Addis Ababa University repository home. Following the removal of duplicate research, 906 studies were selected for full title and abstract screening. Subsequently, 46 studies were screened for full-text articles, after the elimination of 860 studies based on their titles and abstracts. Upon reviewing the full text, 32 articles were excluded for further reasons. Ultimately, this systematic review and meta-analysis study's inclusion criteria comprised 14 articles,^27,36,38,46–56^ which involved 33,936 study participants (Fig. 1).

The studies under consideration utilized a cross-sectional study design. Of the studies included, 13 were cross-sectional studies conducted at health care facilities, while the remaining studies were community based. The geographic distribution of the studies was as follows: six studies were conducted in Ethiopia^36,38,46,48,51,52^ four in Nigeria,^47,50,55,56^ and four in Ghana.^27,49,53,54^ The sample sizes of studies ranged from 262 to 7,661. The prevalence of intention to use postpartum contraception varied from 44.11% to 84.3%. The quality of the studies was assessed using the JBI quality appraisal checklist, which revealed a low risk of bias in majority of the studies (Table 1).

**Table 1.** Characteristics of Studies Included in the Systematic Review and Meta-Analysis of Intention to Use Postpartum Contraceptives in Sub-Saharan Africa

Author (year)	Country	Setting	Design	Sample size	Prevalence	Quality
Idowu (2015)^56^	Nigeria	Institutional	Cross-sectional	444	65%	Low risk
Eliason et al. (2013)^54^	Ghana	Institutional	Cross-sectional	1,914	70%	Low risk
Abraha et al. (2018)^38^	Ethiopia	Community	Cross-sectional	590	84.3%	Low risk
Ujah OI et al. (2017)^55^	Nigeria	Institutional	Cross-sectional	262	64%	Low risk
Eliason et al. (2018)^53^	Ghana	Institutional	Cross-sectional	1,091	78%	Low risk
Gebeyehu et al. (2020)^52^	Ethiopia	Institutional	Cross-sectional	416	70%	Low risk
Adegbola, (2009)^50^	Nigeria	Institutional	Cross-sectional	423	54%	Low risk
Tiruneh et al. (2016)^51^	Ethiopia	Community	Cross-sectional	7,589	44.1%	Low risk
Abraham (2016)^48^	Ethiopia	Institutional	Cross-sectional	420	66.2%	Low risk
Ochejele et al. (2019)^47^	Nigeria	Institutional	Cross-sectional	330	69.1%	Low risk
Wuni et al. (2018)^49^	Ghana	Institutional	Cross-sectional	590	NR	Low risk
Ahuja et al. (2020)^27^	Ghana	Community	Cross-sectional	7,661	52.2%	Low risk
Shitu et al. (2022)^46^	Ethiopia	Community	Cross-sectional	6,555	48.63%	Low risk
Gilano and Hailegebreal (2021)^36^	Ethiopia	Community	Cross-sectional	5,651	44.11%	Low risk

### Meta-analysis

#### Prevalence of intention to use postpartum contraceptive

**FIG. 2.**
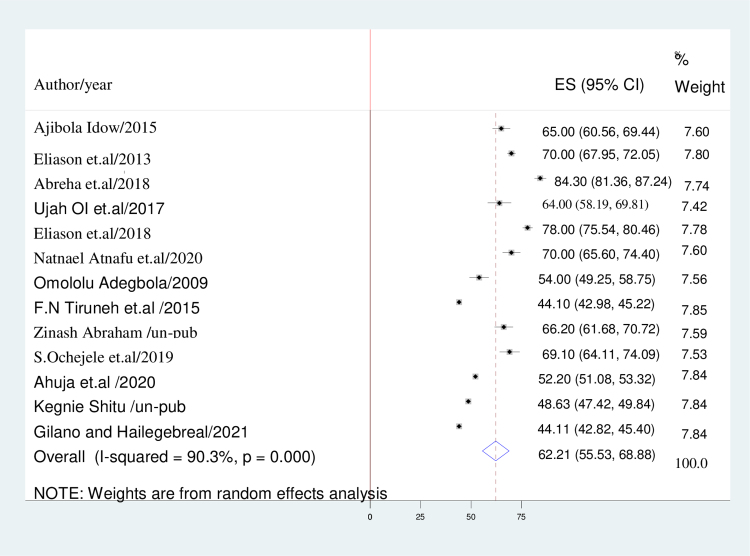
Forest plot displaying the pooled prevalence of intention to use postpartum contraceptives in sub-Saharan Africa.

This study employed a DerSimonian and Laird random-effects model to calculate the aggregate estimate of the intention to utilize postpartum contraception. The heterogeneity index (*I^2^*) was found to be 90.3% (*p* < 0.001), indicating significant variability among the included studies. The pooled prevalence of intention to adopt postpartum contraception among women residing in sub-Saharan Africa was estimated to be 62.21% (95% confidence interval [CI]: 55.532–68.875) (Fig. 2).

#### Subgroup analysis

A subgroup analysis was conducted based on nation, sample size, and study setting due to the significant heterogeneity observed in this meta-analysis. The findings revealed that Ghana had the lowest intention to use postpartum contraceptives (59.39%; 95% CI: 50.22–68.57; *I*^2^ = 72.7%), while Ethiopia had the highest intention (66.71%; 95% CI: 50.36–83.05; *I*^2^ = 87%). The prevalence of the intention to use a postpartum contraceptive in studies conducted in institutions and the community was (67.22%; 95% CI: 62.37–72.07; *I*^2^ = 64.8%) and (54.53%; 95% CI: 46.61–62.45; *I*^2^ = 49%), respectively. Furthermore, studies with sample sizes of less than 1,000 had a prevalence of (56.12%; 95% CI: 47.87–64.36; *I*^2^ = 53%), while studies with sample sizes of more than 1,000 had a prevalence of (67.6%; 95% CI: 59.44–75.25; *I*^2^ = 73.5%) (Table 2).

**Table 2.** The Overall Estimated Intention to Use Postpartum Contraceptives in Sub-Saharan Africa, 95% Confidence Interval, and Heterogeneity Estimate with a *p*-Value for Subgroup Analysis

Variables	Characteristics	Pooled estimate (95% CI)	***I^2^*** (***p***)
Country	Ethiopia	66.71% (50.36–83.05)	87% (0.000)
	Nigeria	63.66% (56.52–69.47)	85.3% (0.000)
	Ghana	59.39% (50.22–68.57)	72.7% (0.000)
Study setting	Institutional	67.22% (62.37–72.07)	64.8% (0.000)
	Community	54.53% (46.61–62.45)	49% (0.000)
Sample size	>1,000	67.6% (59.44–75.75)	52% (0.000)
	<1,000	56.12% (47.87–64.36)	47% (0.000)

CI, confidence interval.

#### Heterogeneity and publication bias

To account for the reported heterogeneity of the study, as indicated by an *I*^2^ value of 90.3%, a subgroup analysis was conducted based on country, sample size, and research setting. In addition, a univariate meta-regression was performed, using sample size and year as covariates, to identify the underlying source of heterogeneity. The findings of the meta-regression revealed that the observed variability among studies was not influenced by either sample size or year (Table 3).

**Table 3.** Meta-Regression Analysis of Factors Affecting Between-Study Heterogeneity

Heterogeneity source	Coefficient's	Standard error	***p***
Year	23.84907	202.3069	0.909
Sample size	4.182912	227.9504	0.986

**FIG. 3.**
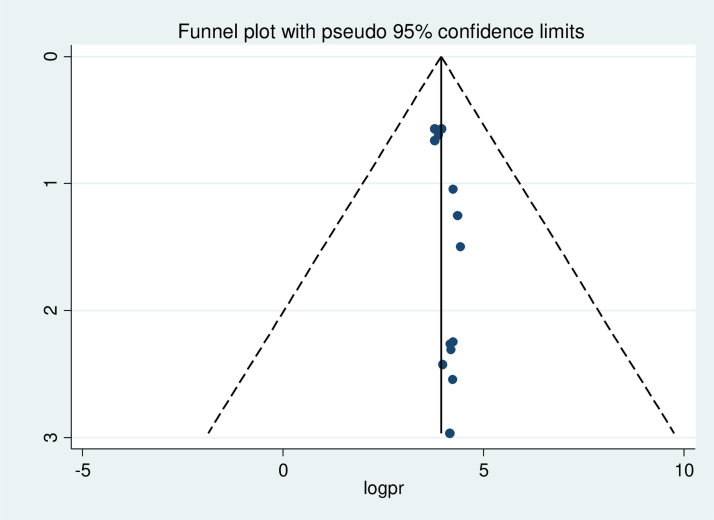
Funnel plot showing asymmetrical distribution of studies indicating absence of publication bias. The Y-axis is the standard error and the X-axis is the study result or effect size. The dotted diagonal lie of the funnel is the 95% CI and the vertical. The vertical line denotes no effect. The square represents the effect size of each study and the line across the square is CI of each study. CI, confidence interval.

**FIG. 4.**
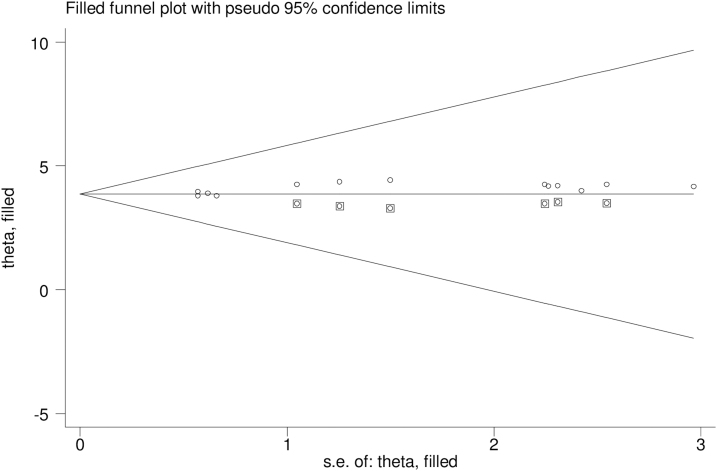
The funnel plot for trim-and-fill method was used to correct the result, six potential missing studies were required in the left side of the funnel plot to ensure symmetry.

In this study, a combination of visual and objective methods was employed to analyze the presence of publication bias. Specifically, a funnel plot was used for visual analysis, while Egger's test and Begg's test were employed for objective assessment. Upon visual observation of the funnel plot (Fig. 3), unequal distribution of studies was observed. The Egger's test (*p* = 0.013) and Begg's test (*p* = 0.031) indicated a significant publication bias, prompting the use of Duval and Tweedie trim-and-fill analysis to address this bias across the studies. As a result of this analysis, the pooled prevalence of intention to use postpartum contraception was revised to 38.67% after the inclusion of six studies and the trim-and-fill analysis. Therefore, the trim-and-fill analysis was used to correct publication bias when six articles were included in the funnel plot (Fig. 4).

**FIG. 5.**
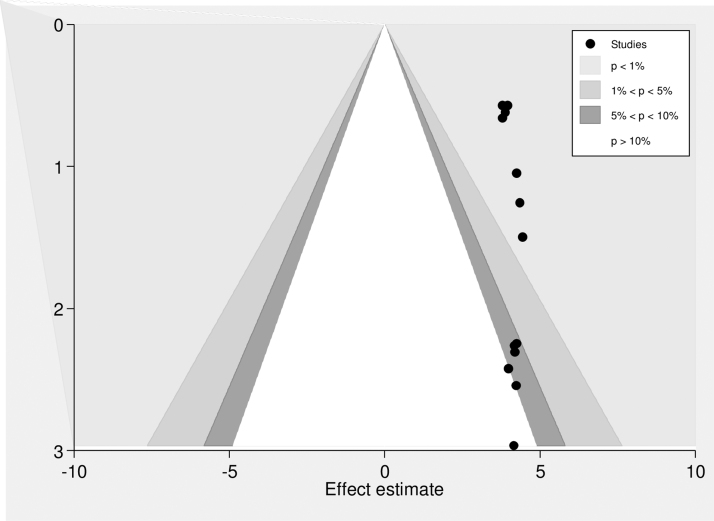
Counter-enhanced funnel plot suggestions of missing studies on the bottom left-hand side of the plot. Since the majority of this area contains regions of high statistical significance (*p* < 0.01), this reduces the plausibility that publication bias is the underlying cause of this funnel asymmetry. Various shaded regions indicate statistical significance. In particular, the white shaded region in the middle corresponds to *p*-values >0.10, the dark gray shaded region corresponds to *p*-values between 0.10 and 0.05, the medium gray shaded region corresponds to *p*-values between 0.05 and 0.01, and the region outside of the funnel corresponds to *p*-values below 0.01.

A counter-enhanced funnel plot was conducted to investigate potential sources of asymmetry in the funnel plot. The results indicate that publication bias is the most probable cause of the observed asymmetry, given that majority of the plot's area comprises regions with significant statistical significance (*p* = 0.01). The plot is divided into different shaded patches to indicate statistical significance, with the center white shaded area corresponding to *p*-values higher than 0.10, and the heavy gray shaded region corresponding to *p*-values between 0.10 and 0.05, the medium gray shaded region corresponding to *p*-values between 0.05 and 0.01, and the area outside the funnel corresponding to *p*-values <0.01 (Fig. 5).

#### Leave-one-out-sensitivity analysis

**FIG. 6.**
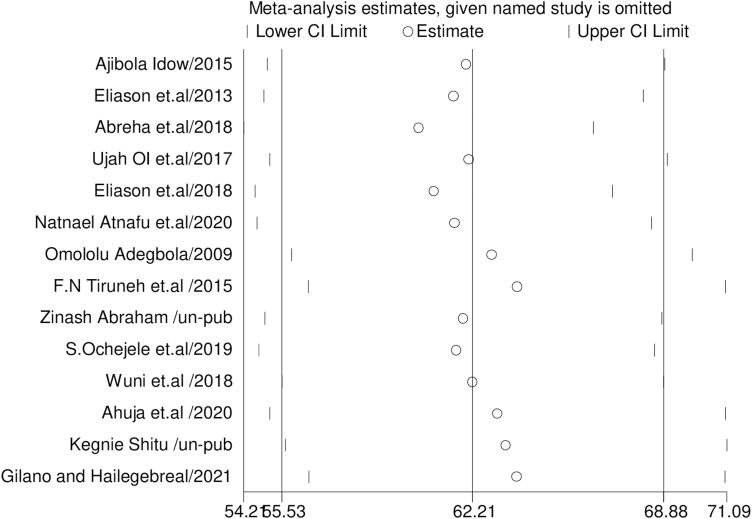
A sensitivity analysis using the leave-one-out method. The exclusion of one study at a time did not yield a statistically significant alteration in the overall prevalence of intention to use postpartum contraceptives.

A sensitivity analysis using the leave-one-out method was conducted to ascertain the impact of individual studies on the overall prevalence of intention to use postpartum contraceptives. The exclusion of one study at a time did not yield a statistically significant alteration in the overall prevalence of intention to use postpartum contraceptives (Fig. 6).

#### Factors associated with intention to use postpartum contraceptive

This research aimed to investigate the potential predictors of intention to use contraceptives, specifically focusing on the variables of primary educational level, prior use of contraceptives, and husband approval of family planning usage. The results indicated that primary educational level and husband approval of postpartum contraceptive use were significantly associated with the use of postpartum contraception. However, no statistically significant relationship was observed between the intention to use postpartum contraceptives and prior use of postpartum contraceptives.

#### Previous history of contraceptive use

**FIG. 7.**
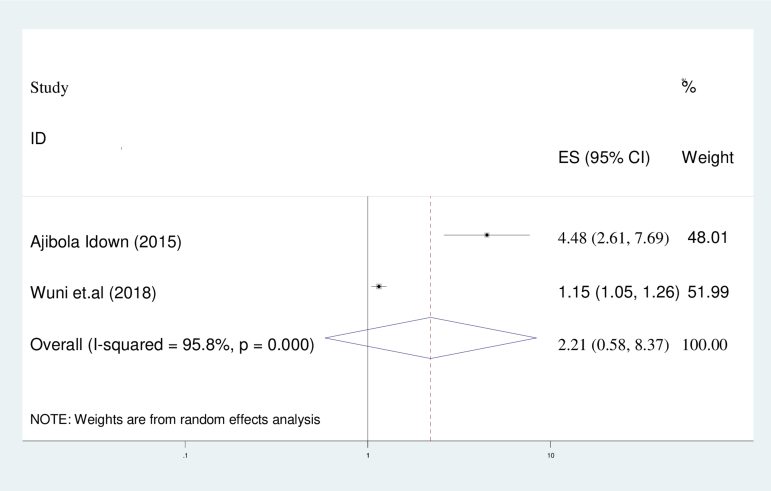
The forest plot of pooled ORs showed the association between previous history of contraceptive use and intention to use contraceptive use with the height of the diamond as the overall effect size (2.21), while the width is the CI at 95% (0.58–8.37). The y-axis shows the standard error of each study, while the x-axis the estimate of effect size of each study. The vertical line denotes no effect. The square represents the effect size of each study and the line across the square is CI of each study. OR, odds ratio.

This study aimed to identify the candidate variables that influence the intention to use contraceptives, namely the primary educational level, prior use of contraceptives, and husband approval of family planning usage. The systematic review and meta-analysis conducted in this study revealed that there is no significant correlation between previous use of contraceptives and intention to use postpartum contraception (adjusted odds ratio [AOR] = 2.21; 95% CI: 0.58–8.37). The analysis employed a random-effect model due to the high level of heterogeneity indicated by the *I*^2^ statistic (95.8%) (Fig. 7).

#### Husband's approval of postpartum contraceptive use

**FIG. 8.**
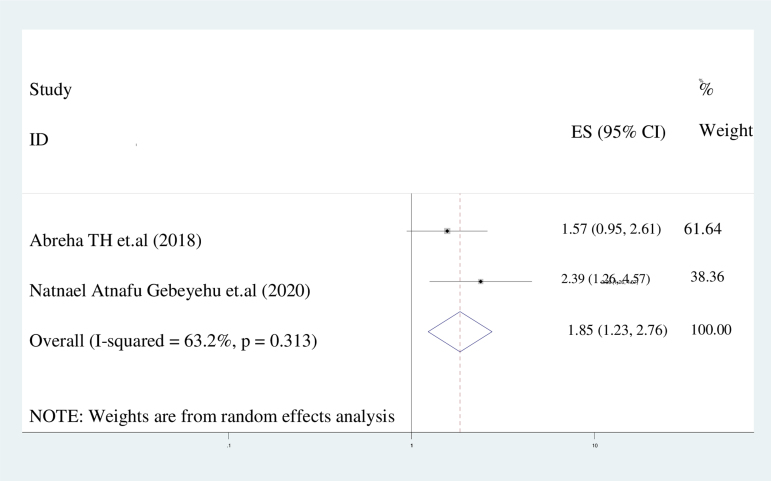
The forest plot of pooled ORs showed the association between husband's approval of postpartum contraceptive use and intention to use contraceptives with the height of the diamond as the overall effect size (2.3), while the width is the CI at 95% (1.256–4.567). The y-axis shows the standard error of each study, while the x-axis the estimate of effect size of the each study. The vertical line denotes no effect. The square represents the effect size of each study and the line across the square is CI of each study.

This meta-analysis has demonstrated that women who secured their partner's consent were twice as likely to express an intention to use postpartum contraception in comparison to those who did not receive such authorization (AOR = 2.3: 95% CI: 1.256–4.567). A random-effects model was utilized, given the *I*^2^ value of 63.2% (Fig. 8).

#### The primary level of education

**FIG. 9.**
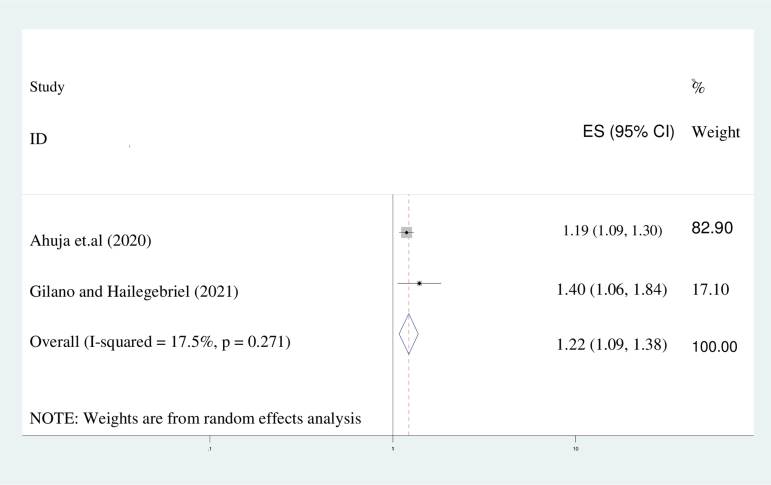
The forest plot of pooled ORs showed the association between primary level of education and intention to use contraceptive use with the height of the diamond as the overall effect size (1.22), while the width is the CI at 95% (1.09–1.38). The y-axis shows the standard error of each study, while the x-axis the estimate of effect size of each study. The vertical line denotes no effect. The square represents the effect size of each study and the line across the square is CI of each study.

This study's results indicate that women who have completed only primary education are 22% more likely to express an intention to use a PPFP method compared to those who have not received any formal education (AOR = 1.22; 95% CI: 1.09–1.38). A random-effect model was utilized due to the presence of heterogeneity among the studies (*I*^2^ = 34.8%) (Fig. 9).

## Discussion

Mothers have the opportunity to initiate family planning methods during the postpartum period; however, it is also a critical time to access family planning services.^57,58^ PPFP services have been advocated as a public health intervention to reduce maternal and child morbidity and mortality rates.^59,60^ The achievement of SDGs^61^ for maternal and child health necessitates an understanding of factors that influence the availability of PPFP options during the postnatal period. The objective of this systematic review and meta-analysis study was to determine the intention of sub-Saharan women to utilize postpartum contraception. The pooled prevalence of intention to use postpartum contraceptives among sub-Saharan women was found to be 62.21% (95% CI: 55.532–68.875), *I*^2^ = 90.3%. This finding is consistent with a study conducted in Bangladesh (65%),^62^ which may be attributed to the similar socioeconomic background of studies.

The findings of this investigation suggest that women who have attained only primary education exhibit a 22% higher likelihood of expressing an intention to employ a PPFP method in comparison to those who have not received any formal education (AOR = 1.22; 95% CI: 1.09–1.38). A random-effect model was employed, given the heterogeneity observed among the studies (*I*^2^ = 34.8%). This investigation has yielded results that surpass those of previous studies conducted in India (27%, 40%),^63,64^ as well as those from the Demographic and Health Survey (40%),^65^ and sub-Saharan African nations (45.7%).^66^ The observed disparity may be attributed to variations in study design, participant age demographics, or the temporal scope of research. For example, a longitudinal study was conducted in India, which focused on married adolescents 15 to 19 years of age.

This study's results indicate a lower percentage than the Ohio State study, which reported a 91% outcome.^67^ The observed disparities between the two investigations may be ascribed to various factors, including socioeconomic conditions, health care system infrastructure, health policies, and participants' level of awareness. In addition, the restricted autonomy afforded to women in sub-Saharan Africa could serve as a further justification for the provision of family planning services.

Following an assessment of the study's study location, study setting, and sample size, a subgroup analysis was performed. As a result, the subgroup analysis focused on the country of origin, revealing that Ethiopia had the highest incidence rate (66.71%) of the intention to use postpartum contraception. Ethiopia has implemented a comprehensive community health program that relies on Health Extension Workers (HEWs) to provide a wide range of community- and facility-based services, including family planning counseling and provision.

The HEWs' activities encompass various services, such as family health, hygiene and sanitation, and reproductive and maternal health, including antenatal care (ANC) and postnatal care (PNC). A study conducted by Mangham-Jefferies et al. revealed that, on average, HEWs allocated approximately one-quarter of their time to family planning and maternal, newborn, and child health activities, which significantly exceeded other health areas.^68^ Furthermore, PPFP counseling is integrated into both ANC and PNC services offered by HEWs and is intended to be integrated into all levels of facility-based maternal care in Ethiopia.^69^ These initiatives demonstrate Ethiopia's commitment to improving the health and well-being of its citizens through a comprehensive and integrated approach to community health.

Research conducted in institutional settings exhibited a higher prevalence of intentions to use postpartum contraception (67.22%) in comparison to studies conducted in community settings (54.53%). This disparity may be attributed to the superior accessibility and knowledge of postpartum contraception among women in institutional studies relative to those in community studies. This phenomenon has a positive impact on women's future utilization of contraception. Furthermore, the prevalence of intention to utilize postpartum contraception was reported to be 56.1% in studies with sample sizes under 1,000, whereas it was 67.6% in studies with sample sizes over 1,000. This trend may be attributed to the fact that the prevalence of postpartum contraceptive intention increases proportionally with the study's sample size.

In this investigation, a significant correlation was observed between the intention to use postpartum contraceptives and the educational level of the mother, as well as the approval of PPFP by the husband. Specifically, women with primary education exhibited a 22% greater likelihood of intending to use postpartum contraception compared to those without formal education. This finding is consistent with prior research conducted across 33 African nations.^66^

The underlying reason for this association is that women who have received formal education are more likely to be exposed to contraceptive options through various media outlets, which enhances their awareness and accessibility to contraceptive alternatives, as well as their ability to comprehend the health benefits of contraception in reducing fertility, unintended pregnancies, unsafe abortions, and other maternal and child health issues.^70,71^ Furthermore, women with higher levels of education possess greater autonomy in their choice of contraception.^72^ These findings suggest that educating women may serve as a viable strategy for increasing the intention to use contraception in these nations.

Upon conducting an extensive search, it has come to our attention that only two studies have been found to report on the approval of husbands, thereby suggesting that our interpretation should be approached with caution. This study reveals that women who obtain their husbands' approval are twice as likely to plan to use postpartum contraception compared to those who do not. This finding is consistent with prior research conducted in sub-Saharan Africa,^73^ which suggests that women in this region have yet to make autonomous decisions regarding contraceptive use.

To address the substantial heterogeneity observed between studies, a random-effect model was employed. The results of a leave-one-out sensitivity analysis indicate that no single study significantly influenced the overall prevalence of intention to use postpartum contraception. Sub-group analysis based on nation, sample size, and publication was conducted to determine the presence of heterogeneity. The observed heterogeneity may be attributed to differences in sample populations, article properties, or sociocultural factors.

## Conclusion

In conclusion, this study found that the prevalence of intention to use postpartum contraceptives was comparatively low in the study population compared to similar studies conducted in other countries. The results indicated that the husband's approval of family planning use and maternal educational status were significantly associated with the use of postpartum contraceptives. Subgroup analysis revealed that Ethiopia had a higher prevalence of intention to use postpartum contraceptives. The findings suggest that women's educational attainment and husbands' approval of family planning are crucial factors that can enhance postpartum contraceptive intentions. Furthermore, it is recommended that the governments of sub-Saharan African nations take a significant share of the responsibility for achieving the 2030 target set by the United Nations to ensure that everyone has access to family planning and other sexual and reproductive health services.

### Limitations and strengths of the study

This study has some limitations. First, articles were restricted to only being prepared and published in the English language. Second, all the included studies were cross-sectional, which might affect the outcome variable because of other confounding factors. Finally, only continuous variables, such as the year and sample size, were included in the meta-regression analysis. This research has also some strengths. First, compressive electronic online international search engines were used. Second, our review incorporated gray literature as part of the primary studies. Third, the predictors for intention to use postpartum contraceptives were discovered.

## Supplementary Material

Supplemental data

Supplemental data

Supplemental data

## Data Availability

All relevant data are within the Manuscript and its Supporting Information files.
