# Supplementary material for: Intention to Use Postpartum Contraceptive and Its Determinants in Sub-Saharan Africa: Systematic Review and Meta-Analysis
Source: Womens Health Rep (New Rochelle). 2023 Dec 15;4(1):627–41. doi: 10.1089/whr.2023.0059 (PMC10754424; doi:10.1089/whr.2023.0059)
Supplement: Supplemental data [file Suppl_TableS2.docx]

Table2. Quality assessment for the included Studies

| Item | Clearly defined inclusion | Describe study setting and participant | Valid and reliable exposure measurement | Objective and standard criteria for measurement | Identified confounder | Strategies to deal with confounders | Valid and reliable outcome measurement | Appropriate statically analysis | No of ‘yes’s ‘ |
| --- | --- | --- | --- | --- | --- | --- | --- | --- | --- |
| Ajibola Idow | Yes | Yes | No | Yes | Yes | No | Yes | Yes | 6/8=75 |
| Eliason et.al | Yes | Yes | Yes | Yes | No | No | Yes | Yes | 6/8=75 |
| Abreha et.al | Yes | Yes | No | Yes | Yes | No | Yes | Yes | 6/8=75 |
| Ujah OI et.al | Yes | Yes | No | Yes | Yes | Yes | Yes | Yes | 7/8=87.5 |
| Eliason et.al | Yes | Yes | No | Yes | Yes | Yes | Yes | Yes | 7/8=87.5 |
| Natnael Atnafu et.al | Yes | Yes | Yes | Yes | Yes | No | Yes | Yes | 7/8=87.5 |
| Omololu Adegbola | Yes | Yes | No | Yes | Yes | Yes | Yes | Yes | 7/8=87.5 |
| F.N Tiruneh et.al | Yes | Yes | Yes | Yes | No | No | Yes | Yes | 6/8=75 |
| Zinashi Abraham | Yes | Yes | No | Yes | Yes | No | Yes | Yes | 6/8=75 |
| S.Ochejele et.al | Yes | Yes | Yes | Yes | Yes | No | Yes | Yes | 7/8=87.5 |
| Wuni et.al | Yes | Yes | Yes | Yes | No | No | Yes | Yes | 6/8=75 |
| Ahuja.et.al | Yes | Yes | No | Yes | Yes | Yes | Yes | Yes | 7/8=87.5 |
| Kegnie Shitu | Yes | Yes | Yes | Yes | No | No | Yes | Yes | 6/8=75 |
| Gilano and Hailegebreal | Yes | Yes | Yes | Yes | Yes | No | Yes | Yes | 7/8=87.5 |
